# Management Diversification Increases Habitat Availability for Lepidoptera Papilionoidea in the Torretes Biological Station (Spain)

**DOI:** 10.3390/insects16070683

**Published:** 2025-06-30

**Authors:** Javier Quinto, Elena Espín, Eduardo Galante

**Affiliations:** Instituto de Investigación CIBIO (Centro Iberoamericano de la Biodiversidad), Universidad de Alicante, 03690 San Vicente del Raspeig, Alicante, Spain; javier.qnt@gmail.com (J.Q.); elenaespinferrando@gmail.com (E.E.)

**Keywords:** protected natural areas, habitat management, butterfly conservation, entomological reserves, Mediterranean ecosystems

## Abstract

Butterflies (Lepidoptera: Papilionoidea), considered bioindicators of habitat conservation status, are one of the groups used to assess the impact of habitat management and improvement efforts in the Biological Station of Torretes (Ibi, Alicante, Spain). This area also houses the Botanical Garden of the University of Alicante. The study was focused on the period from autumn 2022 to late spring 2023, and data on butterfly diversity and abundance from the current study were compared with historical data. Our results highlight that open spaces and the availability of trophic resources (nutritional plants) were key factors influencing the richness and abundance of Papilionoidea. The program for the creation and maintenance of new habitats at the biological station has increased spatial heterogeneity and the availability of trophic resources, which has led to a significant increase in the richness and abundance of butterflies in a short period. To support the conservation of Mediterranean ecosystems, and entomofauna in general, the Papilionidae are proposed as model groups for designing conservation projects based on traditional habitat management. These projects should promote spatial heterogeneity, include programs to enrich plant diversity, and be complemented by environmental education and outreach initiatives.

## Simple Summary

Human intervention has dominated the habitats of the Mediterranean basin since the early Neolithic. Agrosilvopastoral activities have transformed ecosystems, shaping cultural landscapes through human action. This has resulted in a rich spatial mosaic of crop fields and pastures interspersed with fragments of the original forest and native habitats. The abandonment of traditional agrosilvopastoral practices is known to negatively affect biological diversity. The Torretes Biological Station, located in the municipality of Ibi (Alicante, Spain), exemplifies the recovery of the traditional management of a former agricultural estate through a program initiated 21 years ago. This area has witnessed the restoration of habitat diversification and the enhancement of plant diversity, which has positively impacted its biodiversity. To assess the impact of this process on the entomofauna, a study of butterflies, which are considered bioindicators, was conducted to evaluate the effects of habitat management. Our results highlight that the program of creating and maintaining new habitats at the biological station has increased spatial heterogeneity and the availability of trophic resources, leading to a significant increase in the richness and abundance of diurnal butterflies within a short period.

## 1. Introduction

The extinction rate of insect species, both locally and globally, has risen significantly in recent decades, becoming a major scientific concern [1]. Highlighting this issue, the Manifesto of Iberian Entomologists emerged from the XX Iberian Congress of Entomology in June 2023, urging action to stop this decline and raise public awareness about the vital role of insects in ecosystems (https://xxcongresoie.entomologica.es/, accessed on 1 July 2023). Lepidoptera is one of the most diverse and well-studied orders of insects, which has historically aroused both scientific and social interest [2]. The extensive knowledge about their biology and diversity has fueled numerous studies in ecology, evolution, and conservation [3]. Their key ecological roles and sensitivity to environmental changes make them excellent bioindicators [3,4,5], which has strengthened their value as a “star group” for promoting biodiversity conservation and education programs [2]. Within Lepidoptera, the Papilionoidea have received special attention due to their captivating beauty, easy observation, and well-known taxonomy. This superfamily encompasses six families in Europe: Hesperiidae, Lycaenidae, Nymphalidae, Papilionidae, Pieridae, and Riodinidae [6,7]. Many representatives have been identified as crucial pollinators of natural ecosystems and agricultural crops [8] and bioindicators of environmental health [9,10].

The Iberian Peninsula, a biodiversity hotspot in Europe [11,12], boasts a rich mosaic of habitats shaped by a long history of human activity [12,13,14,15,16,17]. This high spatial heterogeneity contributes to the peninsula’s remarkable butterfly diversity. Over 250 butterfly species have been recorded in Spain, rivalling the numbers found in Italy, Greece, and Turkey [18,19]. Notably, 21 species are endemic to the Iberian Peninsula [1,20].

However, like many other insect groups, butterflies face numerous threats. Landscape alteration and fragmentation, agricultural intensification, infrastructure development and the introduction of invasive species all contribute to butterfly decline [1,7,17,21,22,23,24]. Climate change further exacerbates these threats, particularly in the Mediterranean region, where aridity is increasing, thus altering landscape functioning and displacing species to higher altitudes or towards more northern latitudes [23,25,26,27,28,29].

For this study, the Torretes Biological Station (Located in the municipality of Ibi, Alicante, Spain) was selected to assess how management diversification programs in protected Mediterranean natural areas may drive and enhance diversity patterns, using the butterflies as a response group. Located in the municipality of Ibi and managed by the CIBIO Research Institute (Centro Iberoamericano de la Biodiversidad) of the University of Alicante, this natural area has become an important space for biodiversity conservation and environmental dissemination through a land stewardship agreement between the Ibi City Council and the CIBIO Institute. In view of its exceptional biodiversity, this enclave was recognized in 2019 as an Entomological Reserve by the Spanish Association of Entomology (https://www.entomologica.es/torretes, accessed on 1 July 2023). In addition, the Torretes Biological Station houses the University of Alicante Botanical Garden. All this is indicative of the uniqueness of this natural space.

The objectives of this study were (a) to assess diversity patterns (richness, ecological diversity and composition) of butterflies in Torretes, (b) to identify temporal changes in the butterfly community of Torretes through comparisons with previous studies, and (c) to propose specific actions to improve the management and conservation of Mediterranean habitats.

## 2. Materials and Methods

The study was conducted at the Torretes Biological Station (Spain), a former Mediterranean farm that has been transformed into a site for research, conservation and scientific outreach. The area covers 53 ha, 44 of which are forested areas and the remaining 9 are agricultural land. It is located in the north part of the province of Alicante (Spain), on the southern slope of the Carrascal de la Font Roja Natural Park, between 900 and 1100 m a.s.l. The climate is Mediterranean, with certain continental influence due to the altitude, characterized by cool winters with occasional frosts and short, warm summers. The average annual temperature is 14.8 °C, and the average annual precipitation is 459.5 mm [30]. The station is located between the dry mesomediterranean and subhumid supramediterranean levels, with potential vegetation of Kermes oak (*Quercus coccifera* L.) and deciduous formations in the shadiest points.

**Figure 1:**
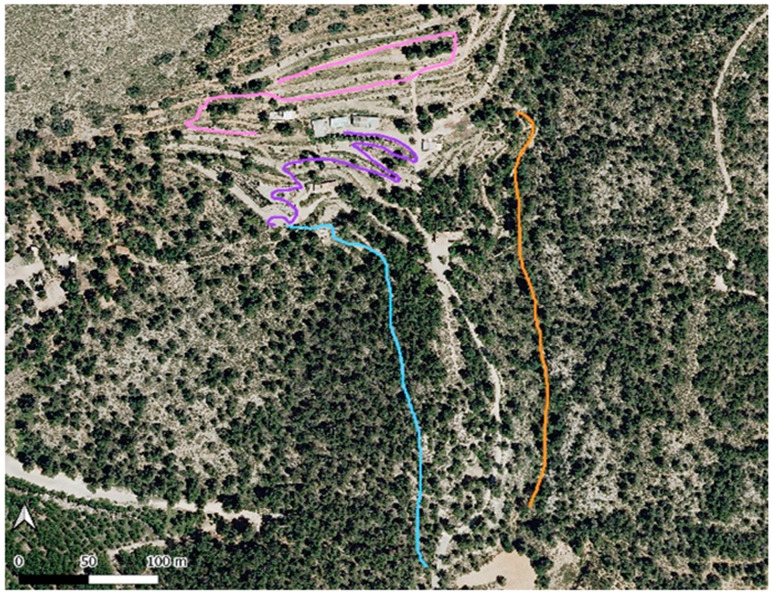
Satellite image of the Torretes Biological Station taken from the National Geographic Information Centre (CNIG), in which the four transects made are delimited with QGIS. Orange: Ravine; pink: Terrace 1; purple: Terrace 2; blue: Pine forest.

**Figure 2:**
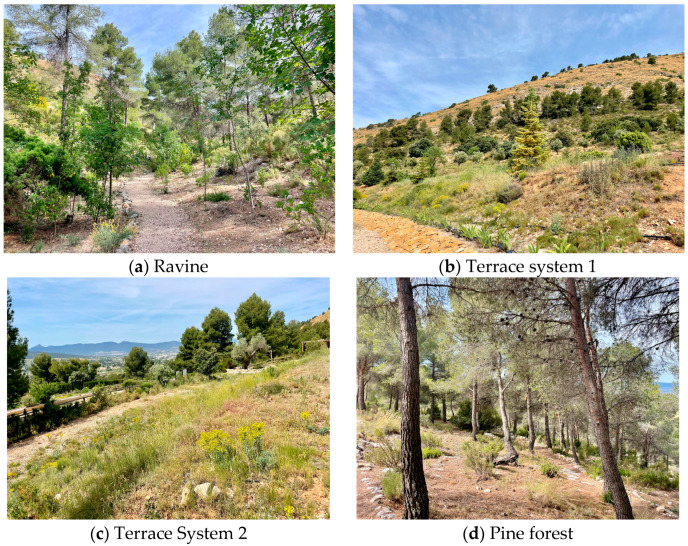
The four habitat types assessed at the Torretes Biological Station.

For this study, the four habitat types present at the Torretes Biological Station [31] were considered (Figure 1):

(a) Ravine (Figure 2a). This habitat, characterized by significant depth and substantial vegetation cover, maintains higher humidity and a lower average morning temperature than the other habitats. It is dominated by a holm oak forest (*Quercus rotundifolia* Lam.) with scrub and scattered specimens of Aleppo pines (*Pinus halepensis* Mill.) and junipers (*Juniperus oxycedrus* L.). Two permanent water points along its course support edaphohygrophilous vegetation such as water lilies (*Ninfea alba* L.) and yellow iris (*Iris pseudacorus* L.).

(b) Terrace system 1 (Figure 2b). This habitat is composed of old cultivation terraces of almond trees (*Prunus dulcis* D.A.Webb) and olive trees (*Olea europaea* L.) that are surrounded by typical Mediterranean scrublands with scattered holm oaks and a small patch of Aleppo pines. This terrace system is part of the Botanical Garden and houses an important collection of lilies (*Iris* spp.) and sages (*Salvia* spp.), as well as gymnosperms (firs, pines, cypresses, yews, cedars, sequoias, etc.). This open space is highly exposed to adverse environmental conditions, especially solar radiation and wind.

(c) Terrace system 2 (Figure 2c). This heterogeneous terrace system includes several buildings, greenhouses and a complex system of plantations, which from its origin was designed to house important botanical collections, with emphasis on typically Mediterranean botanical groups such as Lamiaceae. This is an open space that presents two small ponds with aquatic and edaphohygrophilous plants. This habitat is the heart of the Botanical Garden, where most actions are carried out to promote plant biodiversity

(d) Pine forest. (Figure 2d) This area is dominated by Aleppo pine but includes Mediterranean plant endemism such as Mariola sage (*Salvia blancoana* subsp. *mariolensis* Figuerola), the Alicante lavender (*Thymus moroderi* Pau ex Martínez), or the royal thyme (*Dictamnus albus* L.). Scattered patches of scrubs and some solitary holm oaks are also present.

Species surveys were conducted according to the method of POLLARD & YATES [32]. Transects ranging from 300 and 400 m were established (Figure 1), with a sampling duration of 30 min per transect. If the weather permitted, sampling occurred every 10 days over six months, from October 2022 to mid-May 2023, excluding January and February due to low temperatures and limited flight activity. Sampling was carried out on sunny days with minimal wind, between 8:30 a.m. and 12:00 p.m. solar time, a period of high activity for butterflies.

Three distinct sampling methods were employed to collect data: entomological netting, photographic documentation, and in situ identification. Most recorded butterfly species were readily identifiable in the field. However, when there was uncertainty regarding the identification of certain Hesperiidae, Lycaenidae, or *Leptidea* (Pieridae) species, particularly due to the possible presence of *L. reali*, specimens were collected for laboratory identification.

For statistical analyses, individuals of the genus *Pieris* that could not be identified in flight to the species level were excluded (Table 1) to avoid potential biases in the analysis. Differences in diversity patterns among habitats (spatial variation) were analysed. Sampling coverage was estimated for each habitat (*Ĉm*) [33], which ranges from 0 (zero completeness) to 100% (maximum completeness). To assess spatial changes in diversity patterns, Hill numbers (*^q^D*) of orders ^0^*D* and ^1^*D* were used [34]. The 95% confidence intervals were used to compare *^q^D* values [35]. These analyses were performed using iNEXT v2 statistical software [36,37].

Finally, to analyse differences in species composition among habitats, a permutational multivariate analysis of variance (PERMANOVA) was conducted using the Bray–Curtis index and 999 permutations [38]. Multidimensional scaling (MDS) was employed to visualize the relative positions of samples based on their species composition similarity. Bootstrap procedures were used for this analysis and for plotting 95% confidence ellipses. Both PERMANOVA and MDS were performed using PRIMER v7 software [39].

## 3. Results

A total of 962 individuals were recorded, representing 42 species (Table 1). *Lasiommata megera* and *Leptotes pirithous* were the most abundant species, with a total of 83 and 82 individuals, respectively.

**Figure 3:**
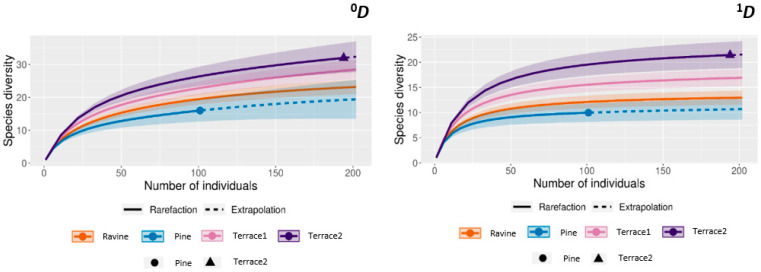
Differences between habitats in species richness (^0^*D*) and ecological diversity (^1^*D*).

Sampling coverage (*Ĉm*) ranged from 0.95 to 0.99 in all habitats studied, indicating adequate sampling effort. Regarding species richness (^0^*D*), significant differences were only found between Ravine and Terrace2 and between Pine forest and Terrace2 (Figure 3). In contrast, significant differences in ecological diversity (^1^*D*) were observed in all habitat combinations)—Ravine vs. Terrace1, Ravine vs. Terrace2, Pine forest vs. Terrace1, Pine forest vs. Terrace2 and Terrace1 vs. Terrace2—except in the case of Ravine vs. Pine forest (Figure 3).

**Figure 4:**
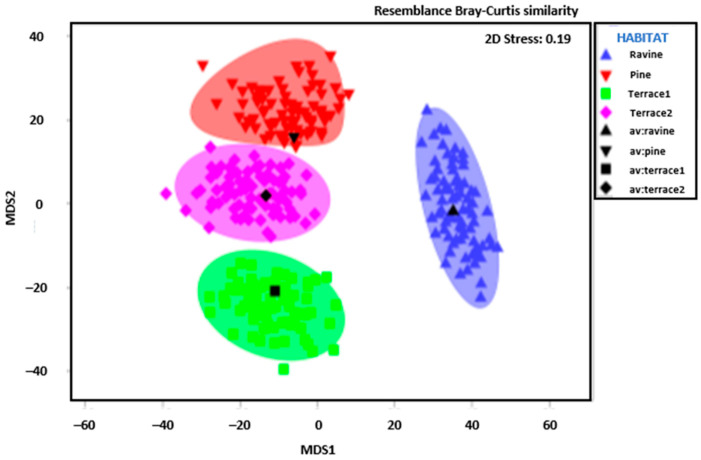
MDS showing the differences in species composition among habitats.

PERMANOVA analysis revealed that all studied habitats (Table S1) exhibited statistical differences in species composition (F_pseudo_ = 2.88, df = 3, *p* < 0.001). No overlap between habitats or ellipses was found in any case (Figure 4). This finding reflects a high level of complementarity among the studied habitats in terms of overall Papilionoid diversity.

## 4. Discussion

The Torretes Biological Station revealed a remarkable biodiversity of butterflies (42 species and 962 individuals) in the period studied between 2022 and 2023. This represents a significant increase in both diversity and abundance compared to a previous study conducted in 2016–2017 (Table S2) using the same methodology and within the same habitats, in which 21 species and 562 individuals were recorded [40]. This relatively small natural protected area encompasses approximately 60% of the butterfly species known in the inland mountain ranges of the northern part of the Alicante province [41,42,43,44]. The richness and abundance of butterflies are strongly influenced by landscape heterogeneity and habitat diversity [45]. These factors provide essential shelter and food resources, including nutritious plants for caterpillars and nectar sources for adults [46,47]. At the Torretes Biological Station, the habitat management and improvement initiatives, coupled with the establishment of the University of Alicante Botanical Garden, have significantly enhanced habitat diversity and plant richness. The management diversification of habitats inherently promotes greater availability of resources, which seems to benefit the overall biodiversity of butterflies in Torretes in the long term.

The highest values of species richness and ecological diversity were observed in the two assessed terraced systems (Figure 3). These managed habitats are characterized by heterogeneous and abundant trophic resources and present extensive sunny areas (low vegetation cover) that are used by many butterfly species for thermoregulation [48,49]. In Terrace 2, the most diverse habitat exhibits a notable presence of butterfly species such as *Pontia daplidice, Colias crocea,* and *Leptotes pirithous*, which are known to prefer open and warm environments [43,50]. Furthermore, the presence of water sources and the diversity of aromatic plant species in these habitats could be contributing to the observed diversity pattern [46,47,51].

In contrast, the ravine and the pine forest habitats presented similar values of richness and ecological diversity. On the one hand, the ravine presented a high number of individuals of certain specialist species (rarely found in the other habitats), such as *Pararge aegeria*, *Goneopteryx cleopatra*, and *Anthocharis euphenoides*, which are associated with more humid environments or the presence of water sources [41,52,53,54,55]. On the other hand, in the pine forest habitat, clearly more homogeneous and less suitable for butterflies [56], no remarkable species were found.

The observed continuous rise in both diversity and abundance of butterflies in less than a decade [40] underscores the critical importance of proper management diversification strategies for natural areas [57]. Since the establishment of the Biological Station in 2003, a program of habitat restoration, management and diversification has been developed and progressively implemented. For instance, this strenuous effort resulted in the creation of the University of Alicante Botanical Garden in 2012 and the Entomological Reserve by the Spanish Association of Entomology in 2019. The management initiatives undertaken in this protected natural area have resulted in the improvement of habitats with multiple trophic resources, fostering biodiversity and transforming it into a reserve renowned for its high Mediterranean entomological diversity [32].

Based on the ongoing habitat diversification and enrichment program at the Torretes Biological Station [31] [Ríos personal communication], we propose improving underrepresented habitats in this area, specifically grasslands dominated by herbaceous plants [58]. Simultaneously, it is crucial to establish areas with diversity of Mediterranean plants that serve as both larval hosts and nectar sources for adult butterflies. This initiative will not only ensure the conservation of existing butterfly populations [58,59] but will also facilitate the potential colonization of additional species documented in nearby areas, such as Font Roja Natural Park [42,60]. These improved spaces will also serve as valuable observation points and platforms for the development of butterfly outreach programs.

Our findings can provide the basis for the design of new educational programs. These could include strategic itineraries with informative displays about butterflies, incorporating details about their food plants and habitats. Such programs have the potential to spark visitor curiosity and significantly raise public awareness about the crucial need to develop and implement insect conservation programs.

## Figures and Tables

**Table 1:** List of species and number of individuals recorded at the Torretes Biological Station in the study period. * *Pieris* sp. refers to the group of individuals recorded in flight of the species *Pieris rapae* and *P. mannii*, as these specimens could not be clearly identified.

Family	Species	Abundance
Hesperiidae	*Carcharodus alceae* (Esper, 1780)	1
	*Erynnis tages* (Linnaeus, 1758)	1
	*Muschampia proto* (Ochsenheimer, 1808)	4
Lycaenidae	*Aricia cramera* (Eschscholtz, 1821)	7
	*Callophrys rubi* (Linnaeus, 1758)	54
	*Celastrina argiolus* (Linnaeus, 1758)	2
	*Glaucopsyche alexis* (Poda, 1761)	4
	*Glaucopsyche melanops* (Boisduval 1828)	8
	*Lampides boeticus* (Linnaeus, 1767)	65
	*Leptotes pirithous* (Linnaeus, 1767)	82
	*Lycaena phlaeas* (Linnaeus, 1761)	1
	*Polyommatus bellargus* (Rottemburg, 1775)	3
	*Polyommatus icarus* (Rottemburg, 1775)	2
	*Pseudophilotes panoptes* (Hübner, 1813)	24
	*Satyrium spini* (Denis y Schiffermüller, 1775)	2
Nymphalidae	*Hipparchia fidia* (Linnaeus, 1767)	7
	*Hipparchia semele* (Linnaeus, 1758)	3
	*Hipparchia statilinus* (Hufnagel, 1766)	3
	*Lasiommata maera* (Linnaeus, 1758)	8
	*Lasiommata megera* (Linnaeus, 1767)	83
	*Maniola jurtina* (Linnaeus, 1758)	2
	*Melanargia ines* (Hoffmannsegg, 1804)	4
	*Melanargia occitanica* (Esper, 1793)	1
	*Melitaea deione* (Geyer, 1832)	6
	*Melitaea phoebe* (Denis y Schiffermüller, 1775)	27
	*Nymphalis polychloros* (Linnaeus, 1758)	3
	*Pararge aegeria* (Linnaeus, 1758)	73
	*Pyronia bathseba* (Fabricius, 1793)	70
	*Vanessa atalanta* (Linnaeus, 1758)	1
	*Vanessa cardui* (Linnaeus, 1758)	29
Papilionidae	*Iphiclides feisthamelii* (Duponchel, 1832)	11
	*Papilio machaon* Linnaeus, 1758	8
	*Zerynthia rumina* (Linnaeus, 1758)	1
Pieridae	*Anthocharis euphenoides* Staudinger, 1869	42
	*Euchloe crameri* (Butler,1869)	3
	*Colias croceus* (Geoffroy, 1785)	55
	*Gonepteryx cleopatra* (Linnaeus, 1767)	69
	*Leptidea sinapis* (Linnaeus, 1758)	7
	*Pieris brassicae* (Linnaeus, 1758)	32
	*Pieris mannii* (Mayer, 1851)	8
	*Pieris rapae* (Linnaeus, 1758)	16
	* *Pieris* sp. Schrank, 1801	61
	*Pontia daplidice* (Linnaeus, 1758)	69
	Total	962

## Data Availability

The data presented in this study are openly available in https://rua.ua.es/dspace/simple-search [RUA, Repository of the University of Alicante].

## Supplementary Materials

The following supporting information can be downloaded at https://www.mdpi.com/article/10.3390/insects16070683/s1. Table S1: Butterfly abundance by habitat, years 2022 and 2023. Table S2: Monthly butterfly flight records. Previous records published in 2016 and 2017 [43] and records provided in this work during 2022 and 2023. The dot indicates a positive record.
