# Supplementary material for: Management Diversification Increases Habitat Availability for Lepidoptera Papilionoidea in the Torretes Biological Station (Spain)
Source: Insects. 2025 Jun 30;16(7):683. doi: 10.3390/insects16070683 (PMC12294853; doi:10.3390/insects16070683)
Supplement: Supplementary file 1 [file insects-16-00683-s001.zip › Table S1.pdf]

Family	Species	Ravine	Terrace1	Terrace2	Pine	Total specimens
**Hesperiidae**	*Carcharodus alceae* (Esper, 1780)	0	1	0	0	1
	Erynnis tages (Linnaeus, 1758)	1	0	0	0	1
	Muschampia proto (Ochsenheimer, 1808)	0	2	2	0	4
**Lycaenidae**	*Aricia cramera* (Eschscholtz, 1821)	0	0	7	0	7
	Callophrys rubi (Linnaeus, 1758)	25	12	11	6	54
	Celastrina argiolus (Linnaeus, 1758)	0	0	0	2	2
	Glaucopsyche alexis (Poda, 1761)	2	2	0	0	4
	Glaucopsyche melanops (Boisduval 1828)	0	7	1	0	8
	Lampides boeticus (Linnaeus, 1767)	14	19	13	19	65
	Leptotes pirithous (Linnaeus, 1767)	11	32	18	21	82
	Lycaena phlaeas (Linnaeus, 1761)	0	0	1	0	1
	Polyommatus bellargus (Rottemburg, 1775)	1	2	0	0	3
	Polyommatus icarus (Rottemburg, 1775)	0	1	1	0	2
	Pseudophilotes panoptes (Hübner, 1813)	5	9	9	1	24
	Satyrium spini (Denis y Schiffermüller, 1775)	0	0	2	0	2
**Nymphalidae**	*Hipparchia fidia* (Linnaeus, 1767)	1	3	3	0	7
	Hipparchia semele (Linnaeus, 1758)	0	0	3	0	3
	Hipparchia statilinus (Hufnagel, 1766)	0	0	3	0	3
	Lasiommata maera (Linnaeus, 1758)	5	2	1	0	8
	Lasiommata megera (Linnaeus, 1767)	27	18	21	17	83
	Maniola jurtina (Linnaeus, 1758)	0	1	0	1	2
	Melanargia ines (Hoffmannsegg, 1804)	0	2	2	0	4
	Melanargia occitanica (Esper, 1793)	0	1	0	0	1
	Melitaea deione (Geyer, 1832)	0	1	5	0	6
	Melitaea phoebe (Denis y Schiffermüller, 1775)	5	8	14	0	27
	Nymphalis polychloros (Linnaeus, 1758)	3	0	0	0	3
	Pararge aegeria (Linnaeus, 1758)	72	0	1	0	73
	Pyronia bathseba (Fabricius, 1793)	22	23	16	9	70
	Vanessa atalanta (Linnaeus, 1758)	0	1	0	0	1
	Vanessa cardui (Linnaeus, 1758)	4	18	5	2	29
**Papilionidae**	*Iphiclides feisthamelii* (Duponchel, 1832)	2	2	7	0	11
	Papilio machaon Linnaeus, 1758	2	5	1	0	8
	Zerynthia rumina (Linnaeus, 1758)	0	1	0	0	1
**Pieridae**	*Anthocharis euphenoides* Staudinger, 1869	35	5	1	1	42
	Euchloe crameri (Butler,1869)	0	2	1	0	3
	Colias croceus (Geoffroy, 1785)	2	37	11	5	55
	Gonepteryx cleopatra (Linnaeus, 1767)	48	13	3	5	69
	Leptidea sinapis (Linnaeus, 1758)	6	0	1	0	7
	Pieris brassicae (Linnaeus, 1758)	14	7	5	6	32
	Pieris mannii (Mayer, 1851)	4	2	1	1	8
	Pieris rapae (Linnaeus, 1758)	8	3	4	1	16
	**Pieris* sp. Schrank, 1801	33	12	11	5	61
	Pontia daplidice (Linnaeus, 1758)	6	46	13	4	69
**Total indiv.**		358	298	197	106	**962**
